# IS THE LINK BETWEEN HEALTH AND WEALTH CONSIDERED IN DECISION MAKING? RESULTS FROM A QUALITATIVE STUDY

**DOI:** 10.1017/S0266462315000616

**Published:** 2015

**Authors:** Martina Garau, Koonal Kirit Shah, Priya Sharma, Adrian Towse

**Affiliations:** Office of Health Economicsmgarau@ohe.org; Office of Health Economics; United States Agency for International Development; Office of Health Economics

**Keywords:** Societal perspective, Indirect costs, HTA systems, Qualitative research

## Abstract

**Objectives:** The aim of this study was to explore whether wealth effects of health interventions, including productivity gains and savings in other sectors, are considered in resource allocations by health technology assessment (HTA) agencies and government departments. To analyze reasons for including, or not including, wealth effects.

**Methods:** Semi-structured interviews with decision makers and academic experts in eight countries (Australia, France, Germany, Italy, Poland, South Korea, Sweden, and the United Kingdom).

**Results:** There is evidence suggesting that health interventions can produce economic gains for patients and national economies. However, we found that the link between health and wealth does not influence decision making in any country with the exception of Sweden. This is due to a combination of factors, including system fragmentation, methodological issues, and the economic recession forcing national governments to focus on short-term measures.

**Conclusions:** In countries with established HTA processes and methods allowing, in principle, the inclusion of wider effects in exceptional cases or secondary analyses, it might be possible to overcome the methodological and practical barriers and see a more systematic consideration of wealth effect in decision making. This would be consistent with principles of efficient priority setting. Barriers for the consideration of wealth effects in government decision making are more fundamental, due to an enduring separation of budgets within the public sector and current financial pressures. However, governments should consider all relevant effects from public investments, including healthcare, even when benefits can only be captured in the medium- and long-term. This will ensure that resources are allocated where they bring the best returns.

## AIM OF THE STUDY

Traditionally, the primary outcome of health interventions considered by decision makers is the impact on patients’ health in terms of reduced morbidity or mortality. Additionally, interventions can generate “wealth effects” (also referred to as indirect costs, nonhealth benefits, or wider societal effects) which extend beyond the health gains accruing to patients. Wealth effects include: improvements in the labor productivity of patients and of their caregivers; cost savings to healthcare, social care, and other sectors; and increases in national income.

In 2003, David Byrne, the then European Commissioner for Health and Consumer Protection, delivered a speech that focused on the importance of health as a “driver of economic prosperity” for European Union (EU) Member States (1). There is a growing body of research aimed at demonstrating the interdependencies between health and wealth (2–4). However, we are not aware of any published studies of whether the consideration of wealth effects, as defined above, has had an impact on resource allocation decisions in practice. This study examines the extent to which the link between health and wealth has influenced national decision making in a sample of eight countries.

We focused on three types of decision makers: health technology assessment (HTA) agencies which make recommendations about the use and/or public reimbursement of health interventions; Health Ministries that run national health systems and in some cases allocate resources across separate health system components; and Finance Ministries/Treasuries that control the budgets of government departments.

## CONCEPTUAL FRAMEWORK

We began by developing a categorization of potential wealth effects based on the published literature. We identified relevant articles by following up the references in recent reviews and comprehensive analyses of the impact of health on economic growth in high-income countries, labor productivity and other indirect costs in economic evaluations (5–9). We identified further publications by conducting searches of Google Scholar using the keywords and abstract terms from these studies.

**Figure 1.**
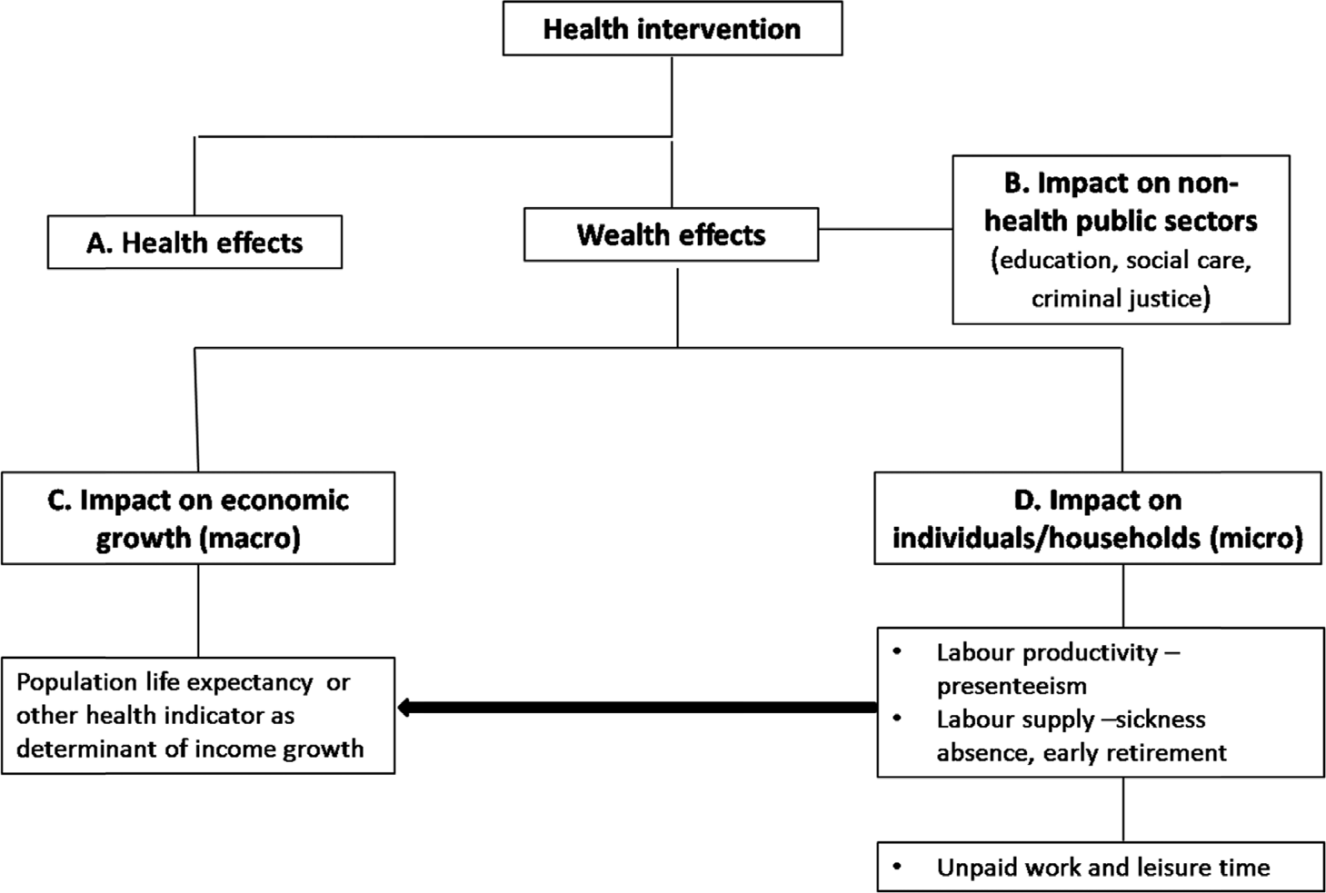
Conceptual framework of the link between health and economic outcomes.

Figure 1 presents our conceptual framework. It illustrates that in addition to health effects such as reducing morbidity or mortality (Figure 1, box A), health interventions can also produce a variety of wealth effects.

The economic costs of illness often fall on sectors other than the healthcare sector; the use of health interventions can lead to important cost savings to those sectors (Figure 1, box B). The resources freed up could then be used to provide additional services within the sector. For example, it has been shown that one of the key drivers of the cost of Alzheimer's disease (almost 40 percent) is the cost of social care provided in patients’ homes or in other community settings (10).

Despite evidence showing that indirect costs can constitute a significant proportion of the total cost of illness to society, the inclusion of those costs in economic evaluations remains limited. Stone et al. (11) found that productivity costs were considered in less than 10 percent of published cost-utility analyses.

Figure 1 also shows that at the macroeconomic level, a positive link may exist between the health of a population and the level of national income (Figure 1, box C). At the microeconomic level, healthcare interventions can have an impact on individuals or households by improving patients’ productivity at work (if they are of working age) and by reducing patients’ and carers’ absences from work due to ill health (Figure 1, box D). The arrow linking macro and micro effects indicates that some micro effects are captured at the macro level, for example, reducing sickness absence can improve individual firms’ production which can also contribute to national income growth. Some effects, however, such as time spent doing unpaid work (e.g., housework), tend only to be captured at the micro level.

Empirical evidence using a global sample of countries has shown that health, measured in terms of life expectancy, is a robust predictor of economic growth (12–15). However, the role of health seems to be stronger in the context of low- and medium-income countries compared with high-income countries, where evidence is limited and shows mixed results. For example, Knowles and Owen (16) found that life expectancy had a minor impact on the economic growth of a sample of high-income countries, while Bhargava et al. (17) found that above a certain level of income per capita in high-income countries, improvements in adult survival rates had a negative impact on growth rates.

The results of these types of studies should be interpreted with caution for two reasons. The first relates to the indicators used to measure population health, which in most studies is life expectancy or adult mortality. While there is wide variation in life expectancy between middle- and low-income countries, there is little variation among high-income countries. As a result, more relevant indicators of health are needed to capture the different levels of health in different high-income countries (4). An example of this is cardiovascular disease (CVD) mortality as used in a study by Suhrcke and Urban (18). They show that a 10 percent increase in CVD mortality among OECD countries reduces the per capita income growth rate by one percentage point. CVD mortality was used as a proxy for health for two reasons. The first was the large disease burden of noncommunicable diseases in OECD countries, CVD in particular. The second was the impact on labor productivity, as CVD affects individuals of working age.

The second reason relates to institutional factors that prevent countries from realizing the positive effects of health improvements. As life expectancy exceeds the retirement age by a growing margin, the old age dependency ratio increases, thus negatively impacting government fiscal stability and, indirectly, economic growth. One way to overcome this would be to increase the retirement age so that the improved health of older people can result in an increase of labor supply and productivity (19). Those policies have already been implemented or are under discussion in several countries.

The literature also explores the issue of casual effect between health and wealth and shows that higher income can increase consumption and provision of goods and services promoting health (6;13). This effect will ultimately reinforce the importance of recognizing the role of improving health outcomes on national income, which can create a “virtual” cycle between health and wealth.

At the micro-economic level, ill health can affect individuals’ participation in the labor force in the short-term, long-term or permanently. This affects individuals’ ability to earn income for themselves and their family, to consume market goods and to engage in leisure activities. A body of literature estimates what are called “indirect costs” to society due to ill health. They include losses due to: (i) Reduced productivity at work (presenteeism): some illnesses, such as back pain and depression (9;20), do not necessarily prevent individuals from attending work but may affect their on-the-job performance; (ii) Sickness absence (absenteeism): individuals who are suffering, recovering from illness, or who are undergoing treatment may require absence from work. For example, it is estimated that a major component of the cost of breast cancer is due to patients’ absence from work due to treatment-related symptoms (21); (iii) Non-employment / early retirement: illnesses that are particularly debilitating may result in individuals being unable to return to work (and, therefore, unable to produce output) on a permanent basis. For example, Kobelt (22) reported that 38 percent of the total cost of multiple sclerosis is due to lost productivity from early retirement.

The effects of ill health also apply to those providing informal (i.e., unpaid) care to patients (23). For example, when children attend hospital appointments, their parents often need to be absent from work to take them to their appointments.

## METHODS

We conducted semi-structured interviews with decision makers and academic experts in eight countries. The aim of the interviews was to explore whether the wealth effects of interventions identified in our conceptual framework represented in Figure 1 are considered by HTA agencies in their health technology evaluations, and by government departments in their budget setting decisions. We also asked about the reasons why these wealth effects were or were not considered. Wealth effects were defined as nonhealth, economic effects generated by the use of health interventions, including impacts on labor productivity and supply, and savings to other sectors.

The potential interviewees invited to participate included individuals representing one or more of the three categories of decision makers (HTA agencies, Health Ministries, Finance Ministries). All were either currently employed by the relevant body or ministry, or local academic experts directly involved in their country's HTA processes and/or in advising their country's Ministry of Health.

The initial geographical scope included countries with established or emerging HTA systems, and near universal health coverage: Australia, Canada, France, Germany, Italy, Poland, South Korea, Sweden, Turkey, and the United Kingdom (UK). The final list of countries was based on whether invitees responded to our request for an interview. These were: Australia, France, Germany, Italy, Poland, South Korea, Sweden, and the United Kingdom.

We developed two questionnaires, one to be used for the HTA or reimbursement decision makers and HTA experts (“Questionnaire for HTA decision makers/experts”; Supplementary Table 1); and the other to be used for the employees of Health and Finance Ministries who had little or no technical knowledge of HTA (“Questionnaire for Ministry of Health/Finance/experts”). The questionnaire for HTA decision makers/experts aimed at exploring whether effects on individuals/households (box D of Figure 1) generated by health interventions matter in the HTA processes of the interviewee's country; and if they are, which types of effects tend to be considered, in which diseases areas they are particularly important, what type of evidence is required to show their impact and what are the key issues encountered. Hypothetical interventions for three conditions were presented to illustrate those effects: Alzheimer's disease, breast cancer, and depression. The case studies were developed using data from recently published cost of illness studies (10;21;24). They focused on drug therapies, because many of the interviewees (particularly the HTA experts) were more familiar with the evaluation of drugs than of other types of health intervention, but it was emphasized to all interviewees that we were interested in the effects of all health interventions. In addition, the final question asked interviewees about the impact of new health interventions on national income (box C in Figure 1) and whether it mattered in the decision-making process they had experience of.

**Table 1.** Categorization of Countries According to the Extent of Consideration of Wealth Effects in Resource Allocation Decisions

	Australia	France	Germany	Italy	Korea	Poland	Sweden	UK
Considers wealth effects regularly							✓	
Considers wealth effects in principle but rarely/never in practice	✓					✓		✓
Does not consider wealth effects within the HTA process or healthcare budget-setting decisions			✓	✓	✓			
Does not currently consider any economic/cost data		✓						

The questionnaire for Ministry of Health/Finance/experts asked interviewees about how any effects of health interventions on nonhealth public sectors (box B of Figure 1) influence budget setting decisions, for example, whether resource transfers are possible when benefits from health spending are captured in other sectors. For the sake of simplicity, this questionnaire included only one case study (the hypothetical intervention for Alzheimer's disease).

Both questionnaires included open-ended questions. This enabled the interviewer to structure the interview by asking predefined questions, but also to pursue additional topics in more depth or to probe for information on themes emerging from the interviewers’ answers. The questionnaires were sent to the interviewees in advance of the hour-long telephone interview. Two researchers were present at all interviews. Summary notes of the interviews were sent to the interviewees for confirmation and correction (if necessary) to ensure that all points made in the discussion were appropriately captured.

The finalized notes from the full set of interviews were reviewed by three researchers (M.G., K.S., and P.S.) who, working independently, summarized the answers in a tabular form, proposed categorizations of countries based on their consideration of wealth effects, and grouped common barriers to the inclusion of wealth effects. In particular, based on answers to two key questions (Are wealth effects mentioned in HTA guidelines/methods guide? Are they considered by your HTA body in practice?), we developed a categorization of countries designed to summarize the impact of wealth effects on their decision-making processes. This categorization is presented in the next section.

Results from those analyses were then discussed and validated, and key themes were agreed, in a group discussion involving all four researchers.

## INTERVIEW RESULTS

We interviewed thirteen individuals from eight countries: seven academic experts and six individuals working (either currently or formerly) for HTA agencies or the Ministries of Health; two individuals from each country were interviewed, with the exception of France, Italy, and South Korea. When the experts stated they had a direct experience or extensive knowledge of the processes of the HTA agencies and/or the Ministries of Health in their countries, we asked them questions related to those topics (suggesting that the HTA/Health Ministry perspectives were represented).

In two countries (Italy, Poland), a Ministry of Finance perspective was represented as the interviewees were able to answer the questions about the allocation of resources among different ministries. In all countries, the Health Ministry and HTA perspectives were represented.

### Do Decision Makers Consider Wealth Effects?

Based on our analysis of the interviewees’ responses (following the approach described in the methods section), we assigned each country to one of four categories: countries that consider wealth effects regularly; countries that consider wealth effects in principle but rarely or never in practice; countries that do not consider wealth effects within HTA; and countries that apparently do not currently consider any economic or cost data when making reimbursement and healthcare budget-setting decisions.

As shown in Table 1, with the exception of Sweden, no country considers wealth effects on a regular basis. In Australia, Poland, and the United Kingdom, although economic evaluations of individual drug interventions submitted to HTA agencies could include wealth effects as part of a secondary analysis, in practice this rarely happens. In Germany, Italy, and Poland there is no scope for including anything other than the direct costs to the healthcare sector and benefits of a new drug. In France, the HTA agency did not consider economic or cost data at the time of our analysis.

At the Finance Ministry level, our two interviewees (from Poland and Italy) emphasized that there is reluctance to consider wider effects of health interventions in their decisions about allocating resources across sectors. Two other interviewees (from the United Kingdom and Australia) referred to national policy reports emphasizing the importance of wealth effects (25;26) but noted that these have not resulted in any specific policy changes to date.

### Key Barriers for the Inclusion of Wealth Effects

Our interviews revealed several legislative, evidence, and policy barriers to incorporating wealth effects into decision making. We have grouped those into the following themes: (i) System fragmentation, including a persistent culture of silo budgets whereby interlinks between governmental departments’ expenditures are not considered regularly if at all and views that the healthcare system should concentrate on health; (ii) Methodological and data generation issues, such as difficulties in demonstrating with reliable data the impact of a specific treatment on productivity; (iii) Practical issues due to added complexity if those effects are included in decision making; (iv) Equity issues as the inclusion of productivity effects can favor interventions for working-age individuals; (v) Weakness of evidence on the relationship between health and economic growth at the macro level which is limited in relation to high-income countries.

### System Fragmentation

The general view among decision makers is that the primary and often sole objective of health care is to improve citizens’ health. Thus healthcare budgets tend to be separate from budgets for other sectors even when they are closely related, such as social care. Any spill-overs that occur across sectors are not captured, for example, where spending on a healthcare intervention leads to lower social care costs that are paid out of a separate budget.

In Australia, Italy, and Poland we found that there are also silo budgets within the heathcare sector. In Australia for example, hospital and primary care are financed separately with no scope for transferring any cost savings between the different parts of the healthcare system.

In South Korea, the Government created a separate budget to cover the cost of care for dementia. However, this budget covers community care but not drug costs, which are funded by means of the health budget. Any savings that may result from a new dementia drug that delays the need for community care would, therefore, not be considered in a drug benefit assessment as they would accrue outside the healthcare sector.

In Sweden, even though the HTA body adopts a societal perspective when making reimbursement recommendations on new medicines (i.e., all relevant costs and benefits associated with a treatment and illness are considered), individual County Councils can restrict use of HTA-approved medicines to meet their own budget targets (the key criterion for their decisions is budget impact) (8).

A few examples of integrated decision making, where nonhealth programs recognize health benefits, were identified (for example, local authority-funded cycle lanes in the United Kingdom). However, our interviewees could not identify any cases where nonhealth benefits of medicine-based interventions were taken into account when allocating resources to the healthcare sector or more specifically to the budget for pharmaceuticals.

### Methodological and Data Generation Issues

When incorporating wealth effects in economic evaluation, there are methodological issues around measuring, and providing evidence of, productivity effects. First, there is no methodology to disaggregate productivity gains and improvements in quality of life measured by the quality-adjusted life-years (QALY). Are changes in the individuals’ ability to earn income reflected in the QALY? If they are, there is a potential for double counting those effects.

Second, even when productivity effects are included in the cost-effectiveness estimation of drug interventions (as indirect costs), HTA bodies require evidence showing productivity effects which are directly attributable to the intervention, which is rarely available. For example, what is the proportion of patients that return to work due to the treatment?

In addition, it was noted that short-term absences from work do not necessarily lead to significant losses for the firm employing the patient as the returning employee might catch up on her/his work and be more productive.

Those concerns were highlighted by interviewees from Australia and the United Kingdom, where the HTA process rely on cost-effectiveness evidence. In Sweden, where wealth effects are considered on a regular basis, an interviewee raised concerns about the poor quality of the studies showing productivity benefits underpinning recent submissions to the HTA body. The reason identified was that other HTA bodies such as NICE do not ask for this evidence, hence it is not a priority for companies to collect it. Overall, it emerged that, if HTA bodies were to consider productivity effects and other wealth effects of health interventions, including savings falling to other public sectors, then robust data showing those effects would be demanded.

The interviewees from Poland and South Korea discussed the issue of transferability of the data on indirect effects across countries, as evidence collected in the United Kingdom or Sweden, for example, may not be applicable to them. Therefore, the lack of country-specific data was identified as a barrier to the incorporation of indirect costs in their HTA decisions.

### Practical Issues

Some interviewees were skeptical of the impact that wealth effects, particularly productivity gains, can have on final decisions. As one interviewee stated, indirect costs are unlikely to be “the factor that tips the scale in favor of a treatment or not.”

Furthermore, adopting a wider perspective in economic evaluations would result in more work for HTA agencies and for the manufacturers collecting the evidence. Many of our interviewees questioned whether the inclusion of these wealth effects was worth the additional cost and effort.

In some countries, there are legislative barriers to taking wealth effects into consideration when evaluating health interventions. For example, the National Institute for Health and Care Excellence (NICE) in the United Kingdom has until recently been required to adopt a narrow, healthcare sector perspective as specified in the legislation that defined its remit. The new legislation. Public health is already an exception, partly because many of the actions recommended in public health guidance relate to actors outside the health sector. This is reflected in NICE's public health activities where the Institute is more open to reflecting costs and benefits to other sectors. Similarly, in Poland the objective of the healthcare system is defined by law to be to improve the *health* of the Polish population with no mention of other nonhealth gains. Finally, German decision makers are guided by the statutory Social Code Book regulations, according to which drug benefit assessments should be based on patient relevant benefits identified using clinical endpoints.

### Equity Issues

Including indirect effects in the assessment of health interventions can have distributive effects between different social groups. For example, including productivity effects will favor treatments aimed at working age individuals over those who are unable to work because of permanent disabilities, older/retired individuals (who tend to consume more resources than they produce, although they may have been net producers in the past), and children (who may eventually become net producers, but effects accruing over a life time are difficult to estimate). Importantly, this could result in situations where treatments which extend the lives of the older patients for a certain period of time will be found to be less cost-effective than treatments that extend the lives of working age patients for the same amount of time.

Interviewees from Australia and the United Kingdom had particularly strong concerns around the fact that including productivity effects of health interventions conflicted with the principles of equity and nondiscrimination that their health systems were founded upon. Some of the disadvantaged groups are already among the worst-off in society, so any reprioritization of resources away from them could be deemed to be inequitable.

This is in contrast with the approach in Sweden, which is the only country considering wealth effects on a regular basis, despite the fact that it is not an insurance-based system where, arguably, interventions increasing people's ability to work would be favored by employers contributing to insurance funds.

### Weakness of Evidence on Health Impact on Economic Growth

We asked all interviewees whether the Suhrcke and Urban (18) study, which provides evidence on the impact of improved health outcomes in CVD on macroeconomic growth, had had any resonance in their country. Almost all interviewees said that the study, which was commissioned by the European Commission (4), has not had any impact on their national policy.

There are reservations about applying the Suhrcke and Urban results to inform resource allocation decisions. One issue identified was that the focus of the study is on one disease area, although one with the largest burden in high-income countries. Therefore, the results do not necessarily support investment in healthcare generally as a means to promote economic growth. In addition, the results cannot be used to inform priority setting within the health sector as evidence on the impact of other disease areas on macroeconomic indicators is not available.

A few interviewees questioned the validity of these studies, especially in light of documented methodological limitations (7). Only in the United Kingdom, according to one of the interviewees, was the Suhrcke and Urban (18) study discussed by a decision-making committee; however, this was primarily for public health interventions.

## DISCUSSION

The barriers to the incorporation of wealth effects in decision making identified by our interviewees could be addressed in several ways. Breaking down silo budgeting may be difficult, as this will require not only a change in the operation of government financial systems to allow for resource transfers across departments, but also potentially the need to develop more integrated healthcare systems focusing on outcomes that go beyond health gains.

On the other hand, methodological issues can be addressed in the short term by undertaking research comparing the available approaches (e.g., friction cost approach, human capital approach) to estimating productivity gains and assess their validity in different economic contexts. In addition, empirical studies can be conducted to test the extent to which effects of changes in individuals’ income are captured by the QALY such as the study by Tilling et al. (27). This will give HTA bodies more confidence in considering wealth effects systematically.

A clear signal from HTA bodies to a more open approach to the consideration of wealth effects will encourage bio-pharmaceutical companies to invest in generating the evidence needed to demonstrate the presence and the size of those effects for specific treatments. In particular, for each category of wealth effect, including productivity, there is a need to identify the type of studies that can be undertaken and approach to incorporate this evidence in HTA submissions. If HTA remains ambivalent regarding the importance of wealth effects, companies are unlikely to generate good quality evidence to prove them. The UK Department of Health and NICE recently proposed introducing a new, value-based pricing system for pharmaceuticals (28), based on the recognition that the value of a medicine should capture all benefits to society beyond health. Though the proposal was ultimately rejected, it demonstrates that UK decision makers have at least considered the feasibility of incorporating a broader range of non-health effects in assessment processes.

Equity concerns should be considered in light of certain indirect effects of interventions. Taking into account productivity benefits could result in favoring treatments for diseases affecting individuals of working age. However, all members of society could potentially benefit from keeping people at work if the increased tax revenues are redistributed across different groups (e.g., by means of investment in public services). Furthermore, the improved health of nonworking individuals can also have positive effects on the economy by allowing their (informal) carers to remain in work and maintain their labor supply. This may be particularly true for quality of life-improving treatments for nonworking patients with chronic conditions, whose need for caregiving falls as a result of treatment.

The issue of uncertain results on the link between health and economic growth in high-income countries does not justify moving resources away from the health sector. From a methodological perspective, more research can be done entailing, for example, the use of health indicators other than life expectancy to better reflect variation of health states in rich countries and also the application of the Suhrcke and Urban (18) approach to different disease areas. From a national governments perspective, there is an opportunity to expand taxable income when funding interventions that increase patients’ ability to work and earn income, and, therefore, to set a virtuous cycle of “better health—more income for citizens—more taxable income for governments,” which could increase the total resources available and partly help dealing with public deficits.

## STUDY LIMITATIONS AND SUGGESTIONS FOR FURTHER RESEARCH

Our qualitative analysis was based on a relatively small number of interviews (one or two in each country) conducted by telephone. This was sufficient for us to identify common issues preventing countries from considering all relevant effects of healthcare spending, including positive economic spill-overs. A larger sample, however, would have allowed us to compare a greater number of views and to validate some of the claims being made. Further analyses could include more countries with emerging HTA systems and growing economies (such as Brazil) and new EU Member States facing budgetary pressures, to investigate whether and how health could be considered a long-term investment. In terms of methodology, qualitative approaches other than interviews could be used, such as focus groups or workshops allowing participants to interact with one another and to make recommendations following a period of discussion and deliberation.

## CONCLUSIONS

There is evidence suggesting that, in certain diseases areas, health interventions can produce economic gains for patients and national economies (9;18;22;23). Those benefits include improvements in the productivity of patients and their carers at work, and cost savings to other sectors such as education and social care.

Despite this evidence, the results from our interviews with decision makers and expert commentators in eight countries suggest that, with the exception of Sweden, considerations of the link between health and wealth have little to no impact on decision making, from budget setting across ministries to reimbursement decisions on individual therapies.

In countries with established HTA processes and methods guides that in principle allow the inclusion of wider effects in exceptional cases or secondary analyses (Australia, Poland, and the United Kingdom in our study), it might be possible to overcome some of the methodological and practical barriers identified and move toward a more systematic consideration of wealth effects in drug decision making. The United Kingdom, for example, considered this option when developing a proposal for value-based assessment (28). Ultimately, considering all relevant elements, including both health and wealth effects, is consistent with principles of efficient priority setting and does not necessarily require increasing the healthcare budget.

As far as national government decisions are concerned, barriers to the consideration of wealth effects in decision making and investment assessments are more fundamental due to an enduring separation of budgets within the public sector (and in some cases within the health sector) which prevents the capture of spill-overs across areas. In addition, given current financial pressures, it seems unlikely that governments will be willing to shift their focus away from cost-cutting measures aimed at reducing fiscal deficits in the short term toward public investments, including in healthcare, with longer-term benefits. Governments should not, however, overlook how to make the best use of the available resources and should consider all relevant effects, whether positive or negative, when making resource allocation decisions. In difficult economic times it becomes even more important to use resources where they bring the best returns to the economy.

## Supplementary material

For supplementary material accompanying this paper visit http://dx.doi.org/10.1017/S0266462315000616. click here to view supplementary material

## CONFLICTS OF INTEREST

The work on this paper conducted by Martina Garau, Koonal Shah, Priya Sharma, and Adrian Towse was funded by Eli Lilly and Company. The authors have no other relevant affiliations or financial involvement with any organization or entity with a financial interest in or financial conflict with the subject matter or materials discussed in the manuscript.
