# Supplementary material for: IS THE LINK BETWEEN HEALTH AND WEALTH CONSIDERED IN DECISION MAKING? RESULTS FROM A QUALITATIVE STUDY
Source: Int J Technol Assess Health Care. 2015;31(6):449–56. doi: 10.1017/S0266462315000616 (PMC4824956; doi:10.1017/S0266462315000616)
Supplement: Supplementary file 1 [file S0266462315000616sup.zip › S0266462315000616sup/S0266462315000616sup001.docx]

# Supplementary Table 1. Questionnaire for HTA decision-makers/experts

**Introduction**

Health interventions seek to generate direct health benefits but can also increase economic activity. On this basis, expenditure on ‘health’ can be seen as an investment which generates ‘wealth’.

The purpose of this interview is to explore the extent to which payers and decisions makers in your country take into account the wider ‘wealth’ effects of health interventions in their resource allocation decisions.

The three scenarios presented below are used to illustrate how health interventions can lead to productivity benefits and to savings to other public sectors, in addition to the health sector. The scenarios do not include questions about specific treatments but look at the impact of hypothetical interventions.

**Scenario 1: Alzheimer’s disease**

Alzheimer’s disease is a neurodegenerative disorder and is the most common cause of dementia in the elderly. Currently, there are no curative treatments available for Alzheimer’s but there are therapies that can help slow the progression of the disease. Alzheimer’s is a major cause of morbidity and mortality among the elderly and has high costs of care. It is estimated that the annual cost of care per patient with Alzheimer’s in Sweden ranges from €2,452 to €11,321, depending on the perspective adopted (Jönsson and Berr, 2005).

The main cost components, as set out in the table below, are: medical care (comprising hospitalisation costs, drugs costs and outpatient care costs), community care costs and informal care costs (care given free of cost by family and friends – measured in terms of the lost productivity of caregivers). Together, these last two components make up 77% of the total cost of care.

| **Cost component** | **Cost (€)** | **% of total cost** |
| --- | --- | --- |
| *Direct medical costs* | *2,452* | *21.6%* |
| - Hospitalisation costs | 944 | 8.3% |
| - Drug costs | 1,007 | 8.9% |
| - Outpatient care costs | 501 | 4.4% |
| *Other costs* | *8,869* | *78.3%* |
| - Community care costs | 4,522 | 39.9% |
| - Informal care costs (lost productivity of carers) | 4,222 | 37.3% |
| - Transportation costs | 125 | 1.1% |
| **Total cost of Alzheimer’s disease** | **11,321** | **100.0%** |

Source: Jönsson and Berr (2005).

Suppose that you have to review a new treatment for Alzheimer’s which has been shown to reduce disease progression by 50% for three years. After three years, treatment is stopped and disease progression returns to the same level as that for untreated patients. Jönsson et al. (1999) estimated that in this scenario, there would be reduced need for community care (resulting in a 66% reduction in costs) and informal care (resulting in a 17% reduction in costs). The treatment offers no reduction or increase in other costs compared to current clinical practice.

| **Cost component** | **Current practice (€)** | **With new treatment** |
| --- | --- | --- |
| *Direct medical costs* | *2,452* | No change 🡪 *2,452* |
| *Community care costs* | *4,522* | Reduced by 66% 🡪 *1,537* |
| *Informal care costs* | *4,222* | Reduced by 17% 🡪 *3,504* |
| *Transportation costs* | *125* | No change → *125* |
| **Total cost of Alzheimer’s disease** | **11,321** | **7,708** |

**Scenario 2: Breast cancer**

Breast cancer is the most common form of invasive cancer in women worldwide. Health outcomes for breast cancer patients have improved considerably in recent decades due to breakthrough advances in diagnostic testing, surgical procedures and drug therapy. Given the incremental nature of innovation in this field, many of the recent breast cancer treatments have generated small improvements that have nevertheless continued to lead to important advances in the medicinal management of breast cancer and in patients’ health outcomes. It is estimated that the annual cost per patient with primary breast cancer in Sweden is between €8,629 and €25,989, depending on the perspective adopted (Lidgren et al., 2007).

The main cost components, as set out in the table below, are: direct medical costs (comprising inpatient episode costs, outpatients visit costs, and drug costs), informal care costs (care given free of cost by family and friends – measured in terms of the lost leisure time of caregivers) and indirect costs (driven primarily by patients’ absences from work due to treatment-related symptoms).

| **Cost component** | **Cost (€)** | **% of total cost** |
| --- | --- | --- |
| *Direct medical costs* | *8,629* | *33.2%* |
| - Inpatient episode costs | 2,944 | 11.3% |
| - Outpatient visit costs | 5,065 | 19.5% |
| - Outpatient drug costs | 620 | 2.4% |
| *Informal care costs* *(lost leisure time of carers)* | *1,130* | *4.3%* |
| *Indirect costs (absence from work)* | *16,230* | *62.5%* |
| **Total cost of breast cancer** | **25,989** | **100.0%** |

Source: Lidgren et al. (2007).

Suppose that you have to review a new drug for the treatment of primary breast cancer. The new drug offers similar effectiveness to alternative treatments already used in clinical practice, but has a different side effect profile. More specifically, the new drug reduces the severity of treatment-related adverse events, which means that patients would require 25% less sickness absence than if they took the existing treatment regimens. The treatment offers no reduction in other costs compared to alternative existing treatments.

| **Cost component** | **Current practice (€)** | **With new treatment** |
| --- | --- | --- |
| *Direct medical costs* | *8,629* | No change 🡪*8,629* |
| *Informal care costs* | *1,130* | No change 🡪 *1,130* |
| *Indirect costs* | *16,230* | Reduced by 25% 🡪 *12,173* |
| **Total cost of breast cancer** | **25,989** | **21,932** |

**Scenario 3: Depression**

The WHO predicts that in the next decade, depression will become the second most burdensome disease in the world. It is estimated that the annual cost of depression in Sweden ranges from €502 million to €3,542 million, depending on the perspective adopted (Sobocki et al., 2007).

The main cost components, as set out in the table below, are: medical costs (comprising inpatient care costs, outpatient care costs and drug costs), lost productivity due to sickness-related absence, lost productivity due to early retirement and lost productivity due to mortality. Costs relating to absence from work and early retirement are particularly high – these together account for 79% of the total cost of depression.

| **Cost component** | **Cost (€ m)** | **% of total cost** |
| --- | --- | --- |
| *Direct medical costs* | *502* | *14.2%* |
| - Inpatient care costs | 187 | 5.3% |
| - Outpatient care costs | 219 | 6.2% |
| - Drug costs | 96 | 2.7% |
| *Indirect costs* | *3,040* | *85.8%* |
| - Lost productivity (sickness-related absence) | 1,146 | 32.4% |
| - Lost productivity (early retirement) | 1,659 | 46.8% |
| - Lost productivity (presenteeism) | 235 | 6.6% |
| **Total cost of depression** | **3,542** | **100.0%** |

Source: Adaptation from Sobocki et al. (2007).

Suppose that you have to review a new drug for the treatment of depression. The new drug has been shown to reduce the severity of symptoms, which results in a 5% reduction in lost productivity. All other costs remain the same.

| **Cost component** | **Current practice (€m)** | **With new treatment** |
| --- | --- | --- |
| *Direct medical costs* | *502* | No change 🡪502 |
| *Lost productivity – sick leave* | *1,146* | Reduced by 5% 🡪 1,089 |
| *Lost productivity – early retirement* | *1,659* | Reduced by 5% 🡪 1,576 |
| *Lost productivity – presenteeism* | *235* | Reduced by 5% →223 |
| **Total cost of depression** | **3,542** | **3,309** |

**Questions**

1. Do wealth effects, as illustrated in the scenarios, matter in the decision making process you are involved in or have experience of?

If yes, please answer the following:

1. Why are they deemed relevant?
2. Are any of the productivity effects deemed *particularly* important (productivity loss of patients due to early sickness absence, early retirement, mortality; presenteeism; productivity loss of carers)?
3. Are there any wealth effects other than productivity that are considered important?
4. How influential have wealth effects been in past decisions? In particular,
   1. In which disease areas have they been particularly important?
   2. Can you provide examples of treatments where the presence of wealth effects played a crucial role in the HTA decision? Are there any guidelines specific to the treatment or policy documents which report the rationale for taking those effects into account?
5. What type of evidence is required to show their impact?
6. To what extent is the process for incorporating wealth effects formalised? Are they considered on a regular basis or under exceptional circumstances?
7. What are the key issues/barriers for including wider costs and benefits in the decision making process you are involved in or have experience of? What are the opportunity costs associated with prioritising treatments delivering productivity/non-health effects?
8. What are the key factors and trade-offs that drive decision-making on resource allocation to and within the health sector?
9. Is the health-wealth link recognized in national programs and action plans beyond the health sector?

If no, can you indicate:

1. What the reasons are for not including these effects when reviewing new interventions?
2. Is the current approach for considering wealth effects likely to change in the near future?
3. Does the impact of new health interventions on national income matter in the decision making process you are involved in or have experience of? The European Commission has published a study showing that morbidity and/or mortality of cardiovascular disease can have an impact on economic growth (Suhrcke and Urban, 2010).
4. Are you aware of that report?
5. Has the evidence provided in that report had any resonance in your country?

**References**

Lidgren, M. et al. (2007) Resource use and costs associated with different states of breast cancer. *International Journal of Technology Assessment in Health Care.* 23: 223-231.

Jönsson, B., Berr, C. (2005) Cost of dementia in Europe. *European Journal of Neurology.* 12(S1): 50-53.

Sobocki et al. (2007) The economic burden of depression in Sweden from 1997 to 2005. *European Psychiatry.* 22: 146-152.

Suhrcke, M., Urban, D. (2010) Are cardiovascular diseases bad for economic growth? *Health Economics.* 19: 1478-1496.
