# Supplementary material for: IS THE LINK BETWEEN HEALTH AND WEALTH CONSIDERED IN DECISION MAKING? RESULTS FROM A QUALITATIVE STUDY
Source: Int J Technol Assess Health Care. 2015;31(6):449–56. doi: 10.1017/S0266462315000616 (PMC4824956; doi:10.1017/S0266462315000616)
Supplement: Supplementary file 1 [file S0266462315000616sup.zip › S0266462315000616sup/S0266462315000616sup002.docx]

# Supplementary Table 2. Questionnaire for Ministry of Health/Finance/experts

Introduction

Health interventions seek to generate direct health benefits but can also generate “wealth” benefits which can be captured at different levels of the economy.

Improved health can increase a person’s productivity by reducing presenteeism, i.e. reduced ability to work due to illness. Similarly, improved health can increase labour force participation by:

- reducing time spent out of work due to illness (absenteeism);
- helping people to return to work quickly after falling sick; and
- helping workers avoid early retirement due to illness-related morbidity or permanent departure from the work force as a result of premature mortality.

Additionally, health spending can have an impact on non-health public sectors. For example, a new drug that treats children with behavioural problems will have spill-over effects into:

- the education system because fewer resources will be needed to care for these children; and
- the justice system as the potential for delinquency decreases among this high risk group will be reduced.

The purpose of this interview is to explore the extent to which decision makers in your country take into account the wider ‘wealth’ effects of health interventions in their policy and budget setting decisions.

Please consider the following scenario, which illustrate how new health interventions can generate wealth effects. The scenarios do not include questions about a specific treatment, but explore the impact of a hypothetical intervention.

Scenario: Alzheimer’s disease

Alzheimer’s disease is a neurodegenerative disorder. It is a major cause of morbidity and mortality among the elderly and has high costs of care. It is estimated that the annual cost of care per patient with Alzheimer’s in Sweden is €11,321^^[[1]](#footnote-1)^^.

The main cost components, as set out in the table below, are: medical care (comprising hospitalisation costs, drugs costs, and outpatient care costs), community care costs, and informal care costs (care given free of cost by family and friends – measured in terms of the lost productivity of caregivers). Together, these last two components make up 77% of the total cost of care.

| Cost component | Cost (€) | % of total cost |
| --- | --- | --- |
| *Direct medical costs* | *2,452* | *21.6%* |
| - Hospitalisation costs | 944 | 8.3% |
| - Drug costs | 1,007 | 8.9% |
| - Outpatient care costs | 501 | 4.4% |
| *Other costs* | *8,869* | *78.3%* |
| - Community care costs | 4,522 | 39.9% |
| - Informal care costs (lost productivity of carers) | 4,222 | 37.3% |
| - Transportation costs | 125 | 1.1% |
| Total cost of Alzheimer’s disease | 11,321 | 100.0% |

Source: Jönsson and Berr (2005).

Currently, there are no curative treatments available for Alzheimer’s but there are therapies that can help slow the progression of the disease. Suppose that there is an opportunity to spend public money on a new treatment for Alzheimer’s which has been shown to reduce disease progression for three years. As shown below, the main benefits from treatment are the reduced need for community care (resulting in a 66% reduction in costs) and informal care (resulting in a 17% reduction in costs). The latter is important because it gives the family and friends of the patient greater opportunities to participate in the labour market. The treatment offers no reduction or increase in other costs compared to current clinical practice. Hence, spending money on this treatment would be beneficial, even though the resulting savings are accrued outside the health sector.

| Cost component | Current practice (€) | With new treatment |
| --- | --- | --- |
| *Direct medical costs* | *2,452* | No change 🡪 *2,452* |
| *Community care costs* | *4,522* | Reduced by 66% 🡪 *1,537* |
| *Informal care costs* | *4,222* | Reduced by 17% 🡪 *3,504* |
| *Transportation costs* | *125* | No change → *125* |
| Total cost of Alzheimer’s disease | 11,321 | 7,708 |

Questions

1. Are wealth effects generated by health interventions deemed important in the decision making process you are involved in or have experience of? More specifically,
   1. What are the stated objectives of your national health system and how does the presence of those effects fit?
   2. Is the health-wealth link recognized in national programs and action plans beyond the health sector?
2. How do non-health effects generated by health interventions affect the allocation of financial resources among different ministries/departments? More specifically,
   1. If returns from health spending are captured in other sectors, are resource transfers/compensations between sectors a viable option?
   2. What are the financial constraints you face in a period of global economic crisis? What are the benefits from health interventions that will be given priority because of the current situation, if any?
   3. What are the key factors and trade-offs that drive decision-making on resource allocation to the health sector?

1. Jönsson, B., Berr, C. (2005) Cost of dementia in Europe. *European Journal of Neurology.* 12(S1): 50-53. [↑](#footnote-ref-1)
